# Neonatal Deep Palmar Space Infection: An Unusual Presentation

**DOI:** 10.7759/cureus.38626

**Published:** 2023-05-06

**Authors:** A Rupesh Rao, Hemant Parakh, P Madan Mohan Rao, K Yeswanth Kumar, Ehteshaam Qadeer

**Affiliations:** 1 Pediatrics and Child Health, Hope Children's Hospital, Hyderabad, IND; 2 Neonatology, Hope Children's Hospital, Hyderabad, IND

**Keywords:** neonatal infections, deep palmar space infection, newborn diseases, newborn and child health, hand infection, early onset neonatal sepsis, neonatal infection

## Abstract

Deep palmar space infection is a rare but potentially serious condition in neonates that requires prompt diagnosis and management. We present the case of a neonate who developed a deep palmar space infection on day two of life. The neonate presented with swelling, erythema, tenderness, and limited movement of the affected hand. The diagnosis was confirmed with imaging using ultrasound, which revealed evidence of fluid collection suggestive of an abscess. Surgical drainage of the abscess and appropriate antibiotic therapy resulted in a successful outcome with complete resolution of symptoms and recovery of hand function. This case highlights the importance of early recognition, appropriate diagnostic workup, and prompt surgical intervention for deep palmar space infection in neonates to prevent complications and achieve favourable outcomes. Additionally, infection prevention measures such as maintaining strict aseptic techniques during invasive procedures in neonates should be emphasised to prevent similar infections in the future.

## Introduction

The deep palmar space in neonates is a critical anatomical region located in the palm of a newborn's hand. It is a complex area that contains delicate structures such as tendons, nerves, and blood vessels that play a vital role in the hand's normal function. The deep palmar space is formed by the intricate interplay of various tissues and structures in the developing hand of a neonate, and any disruption or abnormality in this region can have profound consequences for hand function. Understanding the anatomy and significance of the deep palmar space in neonates is crucial for healthcare providers involved in the care of newborns, including paediatricians, neonatologists, and hand surgeons, as it can help in the diagnosis and management of conditions that may affect the hand function of neonates [1].

## Case presentation

This is a case of a full-term male neonate born via lower (uterine) segment Caesarean section (LSCS), which was taken in view of oligohydramnios. On day two of life, the parents noticed that the baby was not moving the left little finger, and on day three, the neonate gradually developed swelling, erythema, and tenderness over the left palm and little finger. The parents also noticed that there was a pustular lesion on the left little finger. The neonate also exhibited decreased movement of the left fifth finger and increased irritability. There was a history of fever since day two of life; there was no history of trauma or known immunodeficiency. With the above complaint, the baby was shown to the local doctor on day two, and pus for culture and sensitivity was sent from the left little finger. The baby was started on intravenous antibiotics (piperacillin, tazobactam, and amikacin) on day two.

During intravenous cannulation on the right hand on day 2, the doctor noticed that pus was coming from the IV insertion site (right hand) and advised an ultrasound of both hands. With the above complaints at 70 hours (day three) of life, the neonate was brought to our hospital for further management. Upon physical examination, the neonate had a temperature of 100.2°F, a heart rate of 166 beats per minute, and an oxygen saturation of 95% in room air. The left palm and fingers were oedematous, warm, and tender to touch, with limited movement of the affected hand. The skin over the affected area appeared erythematous, with signs of fluctuance or abscess formation. The right hand was also oedematous, warm, and tender to touch, with limited movement of the affected hand.

**Figure 1:**
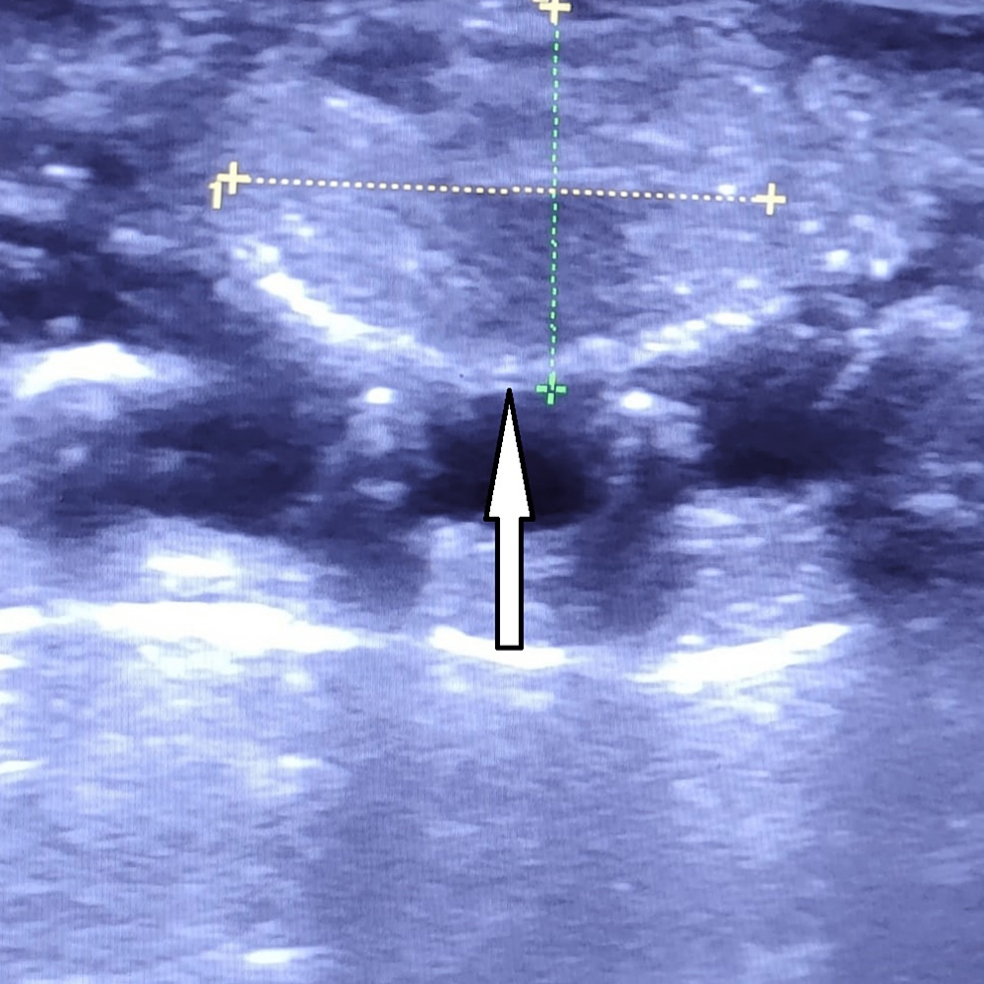
The hand ultrasound showed an abscess in the palmar space which measured 14*12 mm.

Laboratory investigations revealed a haemoglobin of 15.4 gm, a whole blood cell count of 8100 cells/cumm, and a platelet count of 2.4 lakhs/cumm. Blood cultures were obtained, and broad-spectrum intravenous antibiotics were initiated. A hand ultrasound was performed (Figure 1), which revealed evidence of fluid collection in the bilateral deep palmar space, suggestive of an abscess. Based on the clinical findings and imaging results, a diagnosis of deep palmar space infection was made.

**Figure 2:**
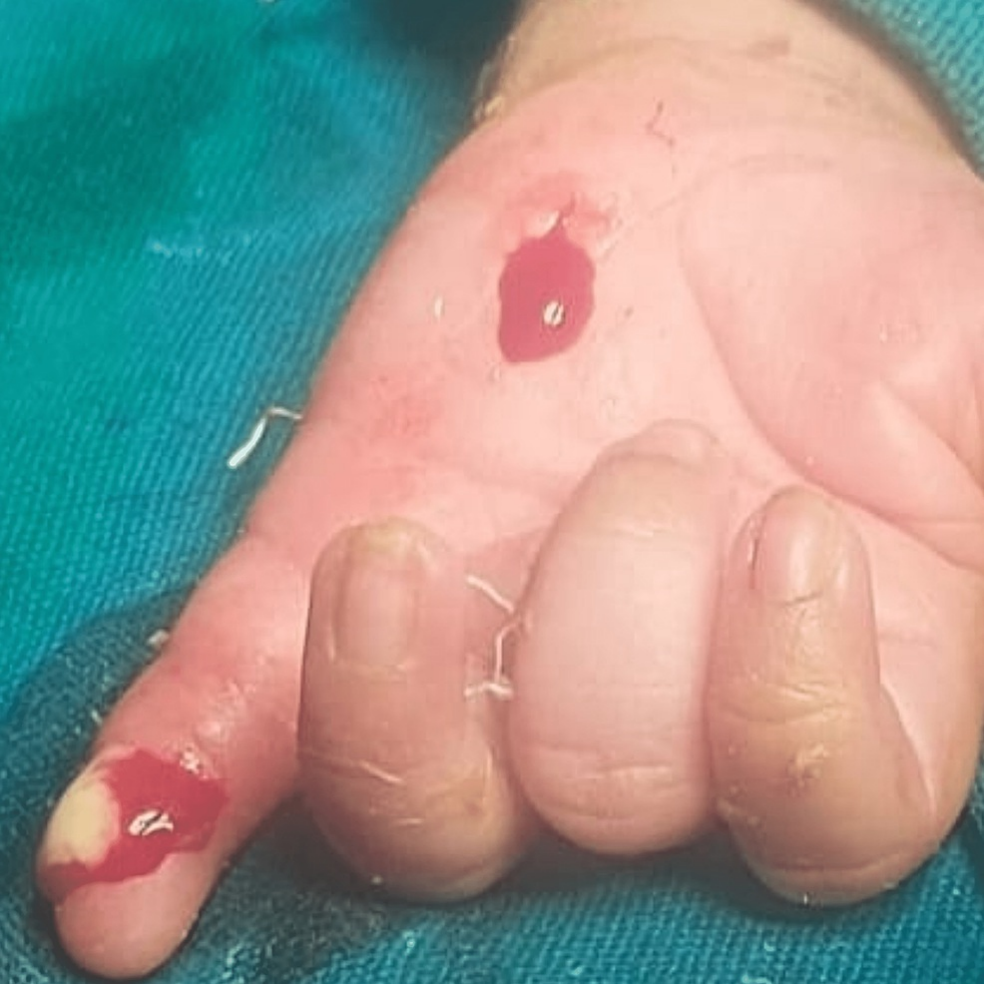
Drainage of pus from the left little finger.

**Figure 3:**
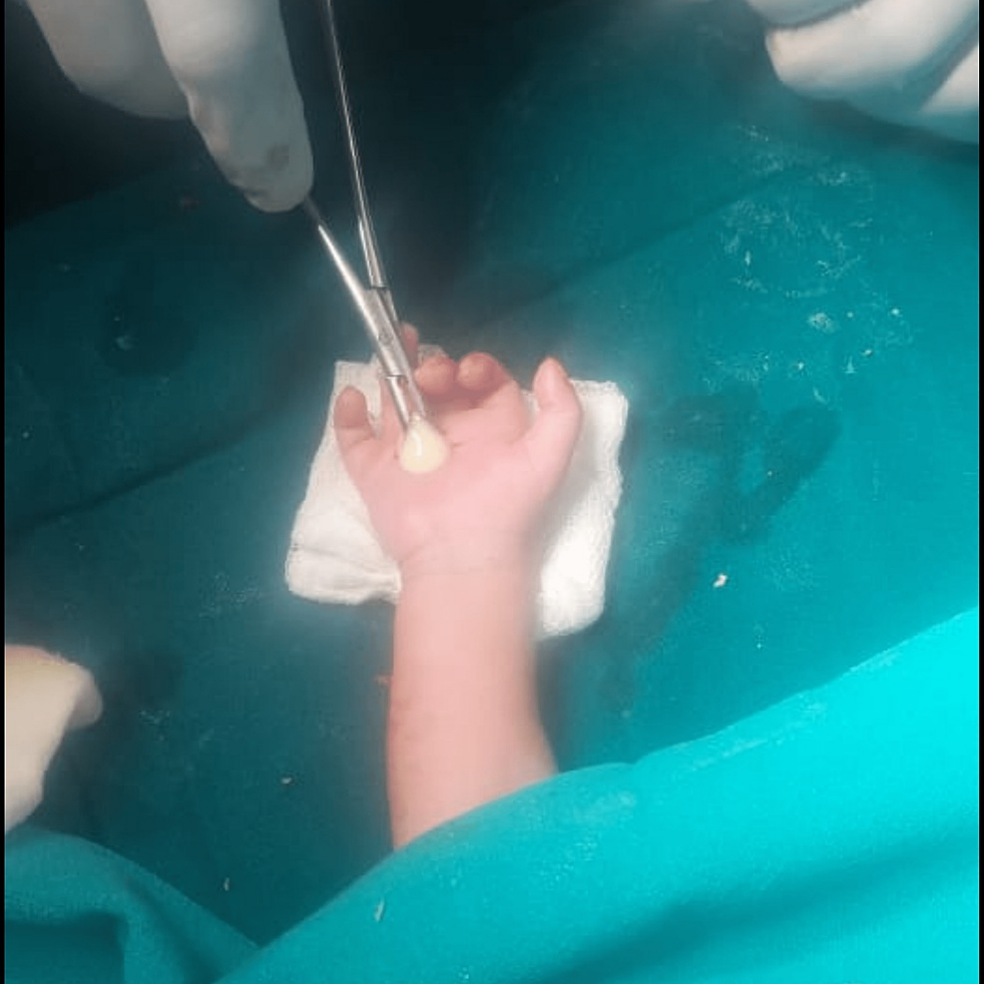
Drainage of pus from the right palmar space.

The neonate underwent surgical drainage of the abscess under general anaesthesia (Figure 2). A longitudinal incision was made in the palm, and pus was evacuated from the deep palmar space (Figure 3).

The pus was sent for culture and sensitivity. Cerebrospinal fluid (CSF) and pus cultures were sterile, but blood cultures showed growth of *Candida *species, therefore an injection of amphotericin B was added based on the culture report. Intravenous antibiotics and antifungals were continued for 14 days. The wound healed well without any complications, and the neonate regained full function in the affected hand.

## Discussion

Deep palmar space infection is a rare condition in neonates that can occur following invasive procedures, such as heel stick blood sampling, or through hematogenous spread from a distant site of infection. The deep palmar space is a confined anatomical space that contains important structures, including nerves, vessels, and tendons, which can be at risk of damage if the infection progresses [2]. Clinical features of deep palmar space infection in neonates can include swelling, erythema, tenderness, limited movement, and systemic signs of infection such as fever and leukocytosis. Early recognition and appropriate diagnostic workup, including imaging with ultrasound or MRI, are crucial in confirming the diagnosis and guiding management [3].

Ultrasound is a non-invasive and readily available modality that can help identify fluid collections suggestive of an abscess [4]. Prompt surgical intervention, such as incision and drainage, is the mainstay of treatment for deep palmar space infection in neonates. Adequate pus evacuation, the culture of the pus, and appropriate antibiotic therapy based on the culture results are important to prevent complications and achieve a favourable outcome. In this case, the neonate had a successful outcome with complete resolution of symptoms and recovery of hand function after surgical drainage and antibiotic therapy. The importance of infection prevention measures, such as maintaining strict aseptic techniques during invasive procedures in neonates, should be emphasised to prevent such infections in the future [5].

## Conclusions

Deep palmar space infection in neonates is a rare but serious condition that requires prompt diagnosis and management. Early recognition of clinical features, appropriate diagnostic workup with imaging, and timely surgical intervention are crucial in achieving favourable outcomes and preventing complications. Infection prevention measures, such as maintaining strict aseptic techniques during invasive procedures, should be emphasised to prevent similar infections in neonates. Healthcare providers caring for neonates should be vigilant in monitoring for signs of deep palmar space infection, especially following invasive procedures, and promptly initiate appropriate interventions to ensure optimal outcomes for these vulnerable patients. Further research and awareness are needed to better understand the risk factors, pathogenesis, and optimal management of deep palmar space infection in neonates.
